# Supplementary material for: Time trends in healthy lifestyle among adults in Germany: Results from three national health interview and examination surveys between 1990 and 2011
Source: PLoS One. 2019 Sep 9;14(9):e0222218. doi: 10.1371/journal.pone.0222218 (PMC6733449; doi:10.1371/journal.pone.0222218)
Supplement: S1 Table — (PDF) [file pone.0222218.s002.pdf]

Supporting Information

S1 Table: Proportions (%, 95%-CI) of individual healthy behavior factors among men aged 25-69 years according to age

Men		1990-92	1997-99	2008-11	Relative change 1990-92 to 2008-11	p#
		% (95% CI)	% (95% CI)	% (95% CI)	%	
**Sufficient physical exercise** [1]		18.5 (16.6-20.5)	20.8 (18.8-23.0)	26.8 (24.5-29.2)	44.9	<.0001
Age	25-34	28.1 (25.4-31.0)	27.9 (24.1-32.0)	36.0 (30.3-42.0)	28.1	0.04
	35-44	18.2 (15.4-21.5)	19.5 (16.4-23.1)	26.8 (22.1-32.1)	47.3	0.007
	45-54	16.6 (13.5-20.2)	20.5 (16.8-24.7)	22.1 (18.6-26.0)	33.1	0.09
	55-69	13.6 (11.1-16.7)	17.3 (14.0-21.3)	24.9 (21.1-29.0)	83.1	<.0001
**No current risk drinking** [2]		46.6 (44.0-49.1)	76.6 (74.5-78.5)	76.8 (74.5-78.9)	64.8	<.0001
Age	25-34	53.5 (49.8-57.1)	72.9 (68.7-76.7)	85.8 (80.9-89.6)	60.4	<.0001
	35-44	40.6 (36.7-44.6)	76.6 (73.2-79.8)	76.7 (71.5-81.2)	88.9	<.0001
	45-54	46.6 (42.8-50.5)	74.9 (70.7-78.7)	71.5 (66.9-75.7)	53.4	<.0001
	55-69	46.6 (42.5-50.7)	80.7 (77.7-83.4)	75.5 (71.7-79.0)	62.0	<.0001
**Daily fruits and daily vegetables** [3]		32.1 (30.3-34.0)	30.8 (28.7-33.0)	19.7 (17.6-22.1)	-38.6	<.0001
Age	25-34	21.4 (18.4-24.8)	20.9 (17.5-24.7)	11.3 (7.5-16.7)	-47.2	0.008
	35-44	28.3 (24.8-32.0)	26.6 (22.9-30.5)	19.0 (14.7-24.3)	-32.9	0.02
	45-54	35.3 (31.7-39.0)	33.6 (29.8-37.6)	19.6 (15.6-24.3)	-44.5	<.0001
	55-69	40.0 (36.5-43.6)	38.8 (35.0-42.8)	26.5 (22.4-31.0)	-33.8	<.0001
**No current smoking** [4]		61.5 (59.4-63.6)	62.3 (59.7-64.9)	65.9 (63.0-68.7)	7.2	0.04
Age	25-34	51.8 (48.3-55.3)	51.0 (46.0-56.0)	49.7 (43.6-55.8)	-4.1	0.85
	35-44	53.7 (49.5-57.8)	52.0 (47.1-56.9)	63.3 (57.8-68.5)	17.9	0.003
	45-54	65.5 (61.9-68.8)	63.1 (58.6-67.3)	64.3 (58.7-69.5)	-1.8	0.70
	55-69	71.3 (67.6-74.7)	78.3 (74.7-81.4)	81.1 (77.9-84.0)	13.7	0.0001
**Normal weight** [5]		29.9 (28.0-32.0)	27.1 (25.0-29.4)	28.7 (26.4-31.1)	-4.0	0.18
Age	25-34	51.0 (47.0-54.9)	43.5 (39.0-48.1)	46.8 (40.8-52.9)	-8.2	0.06
	35-44	32.0 (28.3-36.0)	31.0 (26.8-35.6)	31.8 (27.1-37.0)	-0.6	0.94
	45-54	24.3 (21.1-27.8)	22.5 (18.8-26.5)	23.2 (19.3-27.6)	-4.5	0.77
	55-69	18.6 (15.9-21.7)	16.8 (14.2-19.8)	18.5 (15.8-21.6)	-0.5	0.61

# p for trend
* Prevalences are standardized to the German population as of 31 December 2010.
[1] Sufficient physical exercise: physical exercise ≥2h/week; [2] No current risk drinking: men ≤20g/day; women ≤10g/day; [3] Consumption of fruits and vegetables once or more a day vs. less than daily; [4] No current smoking (never and ex-smoking); [5] Normal weight: BMI 18.5-<25.0 kg/m^2.
