# Supplementary material for: Time trends in healthy lifestyle among adults in Germany: Results from three national health interview and examination surveys between 1990 and 2011
Source: PLoS One. 2019 Sep 9;14(9):e0222218. doi: 10.1371/journal.pone.0222218 (PMC6733449; doi:10.1371/journal.pone.0222218)
Supplement: S2 Table — (PDF) [file pone.0222218.s003.pdf]

Supporting Information

S2 Table: Proportions (%, 95%-CI) of individual healthy behavior factors among women aged 25-69 years according to age

Women		1990-92	1997-99	2008-11	Relative change 1990-92 to 2008-11	p[#]
		% (95% CI)	% (95% CI)	% (95% CI)	%	
**Sufficient physical exercise** [1]		9.9 (8.5-11.4)	14.9 (13.3-16.6)	21.6 (19.7-23.5)	118.2	<.0001
Age	25-34	13.6 (11.2-16.5)	16.2 (13.4-19.6)	19.9 (16.0-24.6)	46.3	0.04
	35-44	10.7 (8.0-14.3)	15.6 (12.8-18.9)	19.0 (15.6-22.9)	77.6	0.006
	45-54	10.6 (8.4-13.2)	15.4 (12.5-18.8)	21.0 (17.8-24.5)	98.1	<.0001
	55-69	6.1 (4.5-8.1)	13.0 (10.6-15.8)	25.3 (21.5-29.5)	314.8	<.0001
**No current risk drinking** [2]		49.3 (47.1-51.4)	71.2 (69.2-73.2)	75.5 (73.1-77.8)	53.1	<.0001
Age	25-34	47.1 (43.4-50.9)	71.4 (67.2-75.2)	88.9 (84.2-92.4)	88.7	<.0001
	35-44	44.9 (40.6-49.4)	72.6 (68.3-76.4)	77.1 (72.5-81.0)	71.7	<.0001
	45-54	46.4 (42.5-50.3)	70.9 (66.7-74.8)	68.6 (63.8-73.0)	47.8	<.0001
	55-69	56.5 (53.1-59.9)	70.4 (66.5-74.0)	71.5 (67.4-75.2)	26.5	<.0001
**Daily fruits and daily vegetables** [3]		53.7 (51.6-55.8)	51.9 (49.4-54.4)	38.0 (35.4-40.6)	-29.2	<.0001
Age	25-34	45.5 (41.8-49.2)	40.7 (35.9-45.7)	29.1 (23.9-34.9)	-36.0	<.0001
	35-44	50.8 (46.6-54.9)	51.1 (46.9-55.3)	36.5 (31.4-41.9)	-28.1	<.0001
	45-54	53.5 (49.7-57.3)	53.8 (48.8-58.7)	40.1 (35.8-44.5)	-25.0	<.0001
	55-69	61.5 (58.0-65.0)	58.1 (53.6-62.4)	43.2 (39.2-47.3)	-29.8	<.0001
**No current smoking** [4]		73.6 (71.7-75.4)	71.5 (69.3-73.6)	70.4 (67.9-72.7)	-4.3	0.10
Age	25-34	57.1 (53.7-60.5)	57.2 (52.3-61.9)	60.0 (54.0-65.8)	5.1	0.69
	35-44	64.6 (60.5-68.6)	62.5 (58.1-66.6)	69.1 (63.7-74.1)	7.3	0.17
	45-54	78.6 (75.4-81.5)	74.5 (70.4-78.2)	68.6 (64.4-72.6)	-12.7	0.0007
	55-69	87.0 (84.5-89.2)	85.0 (82.2-87.4)	79.9 (75.9-83.4)	-8.2	0.003
**Normal weight** [5]		44.2 (41.9-46.5)	43.6 (41.0-46.2)	46.9 (44.4-49.4)	6.1	0.09
Age	25-34	65.8 (62.8-68.8)	63.9 (60.3-67.4)	63.0 (56.8-68.7)	-4.3	0.59
	35-44	55.0 (51.1-58.9)	54.5 (49.4-59.5)	53.2 (48.1-58.1)	-3.3	0.84
	45-54	39.3 (35.5-43.2)	39.0 (34.0-44.3)	45.0 (40.9-49.2)	14.5	0.08
	55-69	25.9 (22.5-29.7)	26.1 (22.8-29.8)	32.7 (29.1-36.6)	26.3	0.007

# p for trend
* Prevalences are standardized to the German population as of 31 December 2010.
[1] Sufficient physical exercise: physical exercise ≥2h/week; [2] No current risk drinking: men ≤20g/day; women ≤10g/day; [3] Consumption of fruits and vegetables once or more a day vs. less than daily; [4] No current smoking (never and ex-smoking); [5] Normal weight: BMI 18.5-<25.0 kg/m^2.

9
