# Supplementary material for: Time trends in healthy lifestyle among adults in Germany: Results from three national health interview and examination surveys between 1990 and 2011
Source: PLoS One. 2019 Sep 9;14(9):e0222218. doi: 10.1371/journal.pone.0222218 (PMC6733449; doi:10.1371/journal.pone.0222218)
Supplement: S1 File — (PDF) [file pone.0222218.s004.pdf]

Supporting Information

S1 File Assessment of alcohol consumption within the German Federal Health Monitoring System

In GNHIES 1990-92, alcohol intake was assessed using the question ‘How often do you consume beverages?’ Participants reported their consumption of beer, wine/sparkling wine, and spirits. The frequency categories were ‘(almost) daily’, ‘several times a week’, ‘about once a week’, ‘2 to 3 times a month’, ‘once a month or less’, ‘never’. The quantity was assessed with the question: ‘How much do you drink on average in one day?’ The categories for beer were ‘more than 2 l’, ‘1 to 2 l’, ‘0,5 to 1 l’, ‘0,25 to 0,5 l’, ‘less than 0,25 l’, ‘(almost) never’; for wine and sparkling wine ‘5 or more drinking glasses (0,25 l)’, ‘3 to 4 glasses’, ‘2 glasses’, ‘1 glass’, ‘less than 1 glass’, ‘(almost) never’; and for spirits ‘10 or more small drinking glasses (0,2 cl)’, ‘5 to 9 glasses’, ‘3 to 4 glasses’, ‘2 glasses’, ‘1 glass’, ‘less than 1 glass’, ‘(almost) never’. In GNHIES 1997-99, data on frequency of alcohol intake during the past 12 months were collected for beer, wine/sparkling wine, and spirits (as in GNHIES 1990-92) using the same frequency categories as in GNHIES 1990-92 except for the daily consumption with two options of ‘daily or almost daily’ and ‘several times a day’. The quantities for wine, sparkling wine, and spirits were exactly the same as in GNHIES 1990-92. Only for beer liters (which is more common) were used in the categories: ‘more than 0,7 l’, ‘between 0,4 and 0,7 l’, ‘between 0,2 and 0,4 l’, ‘between 0,1 and 0,2 l ‘less than 0,1 l’, ‘(almost) never’. In GNHIES 2008-11, frequencies and quantities of beer, wine and sparkling wine, spirits and cocktails and/or mixed alcoholic beverages were also assessed and are already described in detail [1]. In brief, participants reported their frequency of alcohol intake during the past 4 weeks for beer, wine/sparkling wine, and cocktails using the frequency categories ‘more than 5 times a day’, ‘4 to 5 times a day’, ‘3 times a day’, ‘twice a day’, ‘once a day’, ‘5 to 6 times a week’, ‘3 to 4 times a week’, ‘1 to 2 times a week’, ‘ 2 to 3 times a month, ‘once a month or less’, ‘never’. For each survey, frequencies and quantities as well as the mean alcohol content of the specific beverages were used to estimate the average amount of alcohol in gram per day by calculating: frequency × average amount of beverage (l) × alcohol content (g/l))/28 days.

References

1. Lange C, Manz K, Rommel A, Schienkiewitz A, Mensink GBM. Alcohol consumption of adults in Germany. Harmful drinking quantities, consequences and measures. Journal of Health Monitoring. 2016;1(1):2-20. doi: 10.17886/RKI-GBE-2016-029.
