# Supplementary material for: Time trends in healthy lifestyle among adults in Germany: Results from three national health interview and examination surveys between 1990 and 2011
Source: PLoS One. 2019 Sep 9;14(9):e0222218. doi: 10.1371/journal.pone.0222218 (PMC6733449; doi:10.1371/journal.pone.0222218)
Supplement: Attachment — Submitted filename: Review MS Time trends in healthy lifestyle.docx [file pone.0222218.s005.docx]

**Review MS number PONE-D-19-16785**

**Time trends in healthy lifestyle among adults in Germany: Results from three national**

**health interview and examination surveys between 1990 and 2011**

**Abstract**

The abstract is well written and clear.

Methods: Please add e.g. sufficient/ xx amount to ‘Daily fruits and vegetables intake’.

Results: ‘Nevertheless, the gender difference in healthy lifestyle has increased’; Please clarify to which direction. Are men healthier compared to women?

Last sentence; start the sentence with referring to education, so it is directly clear that results for differences in education will be presented.

Conclusions: four out of five vs. 4 or 5 mentioned in the method section.

**Ethics statement**

Did the 1990-92 participants signed an informed consent?

**Introduction**

Line 73-77: Can authors also relate these numbers to a higher prevalence of health related diseases or premature death?

**Materials and Methods**

Line 92-94: Make clear that you compare prevalence data measured at 3 different time points in 3 different cohort. This is not always clear.

Line 104: Mentioning the response rate is not in line with informing that response rates for the three surveys were published previously (line 97).

Line 108 (as mentioned at the ethics statement): Why did participants from the 1997-99 wave did not sign an informed consent?

Line 119: ‘Smoking habits were assessed with the questions allowing a distinction between ‘current smokers’ and ‘others’’. This is a strict cut-off value. Please elaborate why this was so strict. Do authors have any information about former smoking?

Line 114 vs 121: clarify that a healthy diet is defined as fruit and vegetables intake.

Line 123-147: The explanation of alcohol consumption is too wordy which makes it difficult to follow. Recommend to rewrite/shorten or put to supplement.

Line 150: Assessment of fruit and vegetables in 1990-92 is not mentioned to which time period is referred to.

Line 148-168: Restructure, so all information is mentioned and provided in the same order.

Line 170 is repetitive to line 119-120.

The outcome section is well written.

Line 186: add that stratification is also done by gender.

Line 195-197: ‘After exclusion of individuals with missing data for at least one healthy behavior factor used for the HLI, the final study sample consisted of 7,382 participants for GNHIES 1990-92, 5,603 for GNHIES 1997-99 and 5,073 for GNHIES 2008-11’ were there any differences between participants with missing data and the included sample?

Line 207: ‘For age it was standardized to the German population structure as of 31 December 2010.’ To me this is unclear; since you will compare the prevalence of health behavior of people with a certain age (which is fixed since within each cohort you do not check changes over time) in 1990, 1997 and 2008. To what extend does age need to be standardized? Do you mean the results are weighted? And why standardized to 31 December 2010, though data has also been collected in 2011. Please clarify what you mean with this sentence.

**Results**

Authors present the data in the figure stratified by men and women, but why not by age groups or education level. Please elaborate or potentially add visual stratification in the supplement.

Were there any differences between 1990-92 and 1997-99 or between 1997-99 and 2008-11?

Line 235: was the proportion of men with high number of healthy behavior also significantly different.

**Discussion**

Line 271-274 (and line 376): add that trend also include data from 1997-99. Now the impression is that there were only two data collection points.

Line 287-289: “In line with this assumption, the cardiovascular disease mortality has declined in Germany in the last decades and mortality rates are expected to further decline in Germany until 2025 (35-37).” Do authors expect that this is due to a healthy lifestyle or better health care?

Line 306-307: can authors give a potential explanation for this observation? Different legislation?

Limitations

The response rate for GNHIES 2008-11 was much lower (42%) compared to the other two surveys (70% 1990-92 and 61% 1997-99). Please provide some explanation for the discrepancies.

A healthy diet is considered as daily fruit and vegetables intake. What about fibers, and no consumption of sweets and savory products, or soda?

Fruit and vegetable consumption was assessed by means of a self-administered food-frequency questionnaires. Intake was measured for the past 12 months for 1997-99, while it was measured for the past 4 weeks in 2008-11. Acknowledge the inconsistency and provide any expect differences?
